# Navigating the Genetic Landscape: A Comprehensive Review of Novel Therapeutic Strategies for Retinitis Pigmentosa Management

**DOI:** 10.7759/cureus.67046

**Published:** 2024-08-16

**Authors:** Yuga B Pawar, Archana R Thool

**Affiliations:** 1 Ophthalmology, Jawaharlal Nehru Medical College, Datta Meghe Institute of Higher Education and Research, Wardha, IND

**Keywords:** innovative therapies, retinal prosthetics, optogenetics, cell-based treatments, genetic therapies, retinitis pigmentosa

## Abstract

Retinitis pigmentosa (RP) is a collection of retinal disorders characterized by the progressive degeneration of photoreceptor cells, leading to significant visual impairment and, in severe cases, blindness. RP affects individuals worldwide and can be inherited through various genetic patterns, making it a genetically diverse condition. Despite considerable advancements in diagnostic methods and supportive therapies, there is currently no cure for RP. The focus of existing management strategies is on slowing the progression of the disease and improving the quality of life for those affected. This comprehensive review explores the latest therapeutic approaches in the management of RP, highlighting advancements in genetic therapies, such as gene augmentation and editing, as well as cell-based treatments including stem cell transplantation and induced pluripotent stem cell (iPSC) technologies. Emerging methods like optogenetics and pharmacological interventions designed to preserve retinal function are also discussed. Additionally, the review examines technological innovations, including retinal prosthetics and the use of artificial intelligence, which hold the potential to revolutionize RP treatment. The challenges and limitations associated with these novel therapies, such as safety concerns, accessibility issues, and regulatory hurdles, are critically evaluated. By providing an overview of current research and future directions, this review aims to inform clinicians and researchers about the state of the art in RP treatment and the prospects for achieving significant therapeutic advancements.

## Introduction and background

Retinitis pigmentosa (RP) refers to a group of inherited retinal disorders characterized by progressive peripheral vision loss and night blindness, ultimately leading to central vision impairment and, in many cases, complete blindness [1]. The hallmark of RP is the degeneration of photoreceptor cells, particularly the rods responsible for low-light vision, followed by the degeneration of cone cells that mediate color and daylight vision. RP is highly heterogeneous, encompassing a spectrum of genetic mutations that manifest with varying severity and onset [2]. RP is a relatively common cause of inherited blindness, affecting approximately one in 4,000 individuals worldwide. The prevalence varies across different populations and ethnic groups, with some regions exhibiting higher incidences due to factors like genetic drift and founder effects. RP is typically diagnosed in late adolescence to early adulthood, though symptoms can present at any age. The condition impacts both genders equally and can occur sporadically or be inherited in an autosomal dominant, autosomal recessive, or X-linked manner [3].

The genetic landscape of RP is complex, involving mutations in over 60 different genes to date. These genes are critical for the normal function and survival of photoreceptor cells. In autosomal dominant RP, a single copy of the mutated gene is sufficient to cause the disease, often allowing affected individuals to pass the condition to 50% of their offspring [4]. Autosomal recessive RP requires mutations in both copies of the gene, typically leading to the condition manifesting in individuals with unaffected carrier parents. X-linked RP predominantly affects males, as the mutation occurs on the X chromosome, with carrier females usually exhibiting milder symptoms [4]. This comprehensive review aims to navigate the genetic landscape of RP, shedding light on the latest advancements in therapeutic strategies. By exploring novel approaches such as genetic therapies, cell-based treatments, and technological innovations, this review seeks to provide a thorough understanding of the current state and future directions in managing RP. The review will also address the challenges and limitations of these emerging therapies, offering insights into ongoing research and potential breakthroughs that could significantly improve the quality of life for individuals affected by this debilitating condition.

## Review

Pathophysiology of RP

RP is an inherited retinal dystrophy characterized by the progressive deterioration of photoreceptor cells in the retina, resulting in significant visual impairment. Understanding the pathophysiology of RP involves exploring the mechanisms behind photoreceptor degeneration, the impact of genetic mutations, and their effects on visual function and quality of life [5]. The primary mechanism driving photoreceptor degeneration in RP is apoptosis, or programmed cell death, triggered by genetic mutations affecting the rods and cones in the retina. Rod photoreceptors, crucial for low-light vision, are typically more affected than cone photoreceptors, leading to early symptoms like night blindness and peripheral vision loss [6]. As the disease advances, cone cells degenerate, causing central vision loss and impairments in color perception. The degeneration process begins with the buildup of toxic byproducts within photoreceptors, which induces cellular stress and subsequent apoptosis. This cell death can also provoke secondary degeneration in neighboring cells, exacerbating retinal damage. The characteristic "bony spicule" pigmentation observed in RP results from detachment of retinal pigment epithelial cells and pigment deposition in the retina, further complicating the disease's pathology [7]. RP stems from mutations in more than 60 genes, leading to various inheritance patterns such as autosomal dominant, autosomal recessive, and X-linked forms. These mutations disrupt normal phototransduction processes and impair the function of photoreceptor cells [4]. Genetic testing is crucial for pinpointing specific mutations, which can provide insights into prognosis and potential eligibility for gene therapy trials. The genetic diversity of RP results in a broad spectrum of clinical manifestations and rates of progression among affected individuals. While some patients experience rapid vision decline, others may retain functional vision well into adulthood. Identifying specific mutations also offers hope for future therapeutic strategies like gene replacement therapies [8]. The progressive nature of RP profoundly affects visual function, resulting in challenges with daily activities, social interactions, and employment. Initial symptoms often include night blindness and peripheral vision loss, evolving into tunnel vision and eventually central vision loss [9]. This gradual decline in visual acuity significantly impacts quality of life, leading to increased dependence and psychological distress. Individuals with RP commonly face difficulties in mobility, reading, and recognizing faces, contributing to social isolation and diminished well-being. Although rehabilitation programs and low-vision aids can alleviate some challenges, the lack of standardized treatments underscores the critical need for ongoing research and the development of effective therapies [10].

Current diagnostic approaches

**Figure 1:**
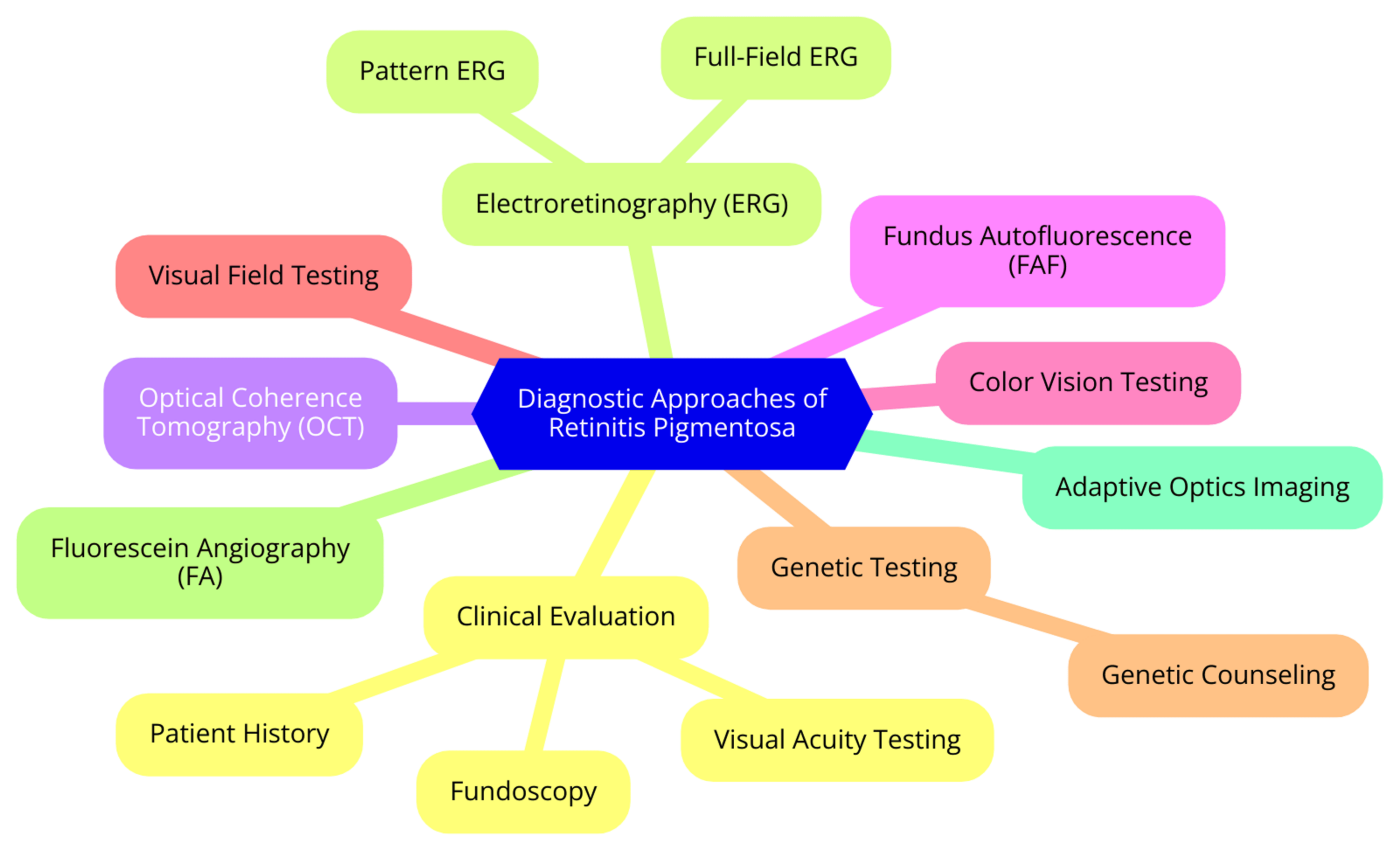
Current diagnostic approaches for RP RP: retinitis pigmentosa Image Credit: Dr. Yuga B. Pawar

RP is a hereditary retinal disorder marked by progressive vision loss, primarily affecting the photoreceptors in the retina. Accurate diagnosis is essential for effective management and potential therapeutic interventions. Current diagnostic approaches include assessing clinical manifestations, utilizing diagnostic tools, employing imaging techniques, and conducting genetic testing [8]. Patients with RP typically present with a range of symptoms that vary widely in severity and onset. Common clinical manifestations include night blindness, which is often one of the earliest symptoms, making it difficult for individuals to see in low light or adjust to darkness. As the disease progresses, patients experience peripheral vision loss, leading to a narrowing of the visual field, often described as tunnel vision [9]. While central vision is usually preserved in the early stages, it deteriorates in advanced RP, resulting in tasks requiring detailed vision, such as reading. Additionally, patients may report visual disturbances, including glare sensitivity and difficulties seeing fine details [11]. Several diagnostic tools and imaging techniques are employed to confirm the diagnosis of RP. Ophthalmoscopy allows clinicians to visualize the retina and assess characteristic changes, such as retinal pigmentary alterations and arteriolar attenuation. The electroretinogram (ERG) is a critical test that measures the electrical responses of photoreceptors to light stimuli. The ERG typically shows reduced or extinguished responses in RP, indicating impaired rod and cone function [5]. Optical coherence tomography (OCT) provides high-resolution images of retinal layers. It can help identify structural changes, such as cystoid macular edema, although it is not primarily used for diagnosing RP. Visual field testing is also important, as it assesses the extent of peripheral vision loss, a hallmark of the condition [12]. Genetic testing plays a pivotal role in the diagnosis and management of RP, particularly given the genetic heterogeneity of the condition. Over 65 genes have been implicated in RP, and genetic testing can identify specific mutations, aiding in diagnosis and informing prognosis [13]. Genetic counseling is essential for understanding the genetic basis of RP, especially for family planning and managing expectations regarding disease progression. Genetic counselors provide valuable information about inheritance patterns and their implications for family members. As gene therapies targeting specific mutations become available, genetic testing will be crucial for determining eligibility for these treatments [14]. Figure 1 illustrates the current diagnostic approaches for RP.

Traditional management strategies

RP management primarily revolves around supportive care, as currently, limited treatment options are available to reverse the condition. One of the most well-researched traditional strategies is vitamin A supplementation. Studies have indicated that taking vitamin A, particularly in the form of palmitate, may slow the progression of vision loss in certain genetic subtypes of RP [15]. The typical recommendation is around 15,000 IU per day. Still, patients must consult with healthcare professionals to monitor dosage and avoid potential toxicity associated with high levels of vitamin A [16]. In addition to vitamin supplementation, various visual aids and adaptive strategies significantly enhance the quality of life for individuals with RP. Low-vision aids, such as magnifiers, telescopic lenses, and electronic visual devices, can help patients maximize their remaining vision. Furthermore, the advent of smartphone applications designed to assist with navigation and reading has provided new avenues for independence [17]. These apps often use auditory feedback to help users interact more effectively with their environment. Additionally, modifications to lighting in living spaces can significantly improve visibility and safety, allowing individuals to navigate their surroundings more confidently [18]. Rehabilitation and support services are essential for helping individuals with RP adapt to their vision loss. Orientation and mobility training is critical, teaching individuals how to navigate their environments safely using techniques such as a cane or other mobility aids [19]. Occupational therapy can also be beneficial, as therapists work with patients to adapt daily activities and work environments to better accommodate their vision limitations. Beyond physical adaptations, psychological support is vital. Counseling services can help address the emotional impact of vision loss, providing coping strategies for patients and their families. Support groups offer a sense of community, allowing individuals to connect with others facing similar challenges and share practical advice on living with RP [19].

Emerging therapeutic approaches

Genetic Therapies

Gene therapy represents a transformative approach to treating genetic disorders, including RP. This strategy encompasses various methodologies, such as gene augmentation and gene editing, which aim to correct or compensate for defective genes. Gene augmentation therapy is primarily used for conditions caused by loss-of-function mutations. This method involves introducing a functional copy of a gene into the patient's cells to restore the production of a necessary protein [20]. For instance, gene augmentation has successfully replaced the missing SMN1 gene in diseases like spinal muscular atrophy to improve patient outcomes. This approach is particularly relevant for RP, where introducing a functional gene can potentially restore photoreceptor function and slow disease progression [21]. Gene editing techniques like CRISPR/Cas9, TALENs, and zinc finger nucleases enable precise genome modifications. These tools can correct mutations at specific sites, providing a more targeted approach than traditional gene augmentation [22]. This method is advantageous as it can address both recessive and dominant genetic disorders by repairing the defective gene or silencing the expression of harmful genes. For example, CRISPR has been utilized in preclinical studies to correct mutations associated with various genetic diseases, including some forms of RP [23]. Numerous clinical trials are currently underway to evaluate the safety and efficacy of gene therapy for RP and other genetic disorders. One of the most notable advancements is Luxturna (voretigene neparvovec), which targets the RPE65 mutation. Clinical trials have shown significant improvements in vision for patients receiving Luxturna, with enhancements in visual function and overall quality of life. This therapy marks a pivotal advancement in gene therapy for inherited retinal diseases [24]. In addition to Luxturna, over 600 clinical trials utilizing somatic cell gene therapy are in progress, focusing on various genetic disorders, including RP. These trials explore the effectiveness of both gene augmentation and gene editing techniques, with early results indicating the potential for significant therapeutic benefits. New gene editing strategies are being developed to address the challenges of traditional gene therapy, such as the need for precise targeting and the avoidance of off-target effects. These innovations are expected to enhance the safety and efficacy of treatments for RP and other genetic disorders [25].

Cell-Based Therapies

Cell-based therapies, particularly those involving stem cell transplantation and induced pluripotent stem cells (iPSCs), are promising strategies for treating RP. These approaches aim to restore retinal function and offer new avenues for managing this degenerative condition [26]. Stem cell transplantation strategies for RP focus on replacing damaged retinal cells and supporting the regeneration of retinal tissue. Various types of stem cells, including embryonic stem cells, mesenchymal stem cells, and iPSCs, are being explored for their potential to differentiate into retinal cell types, such as retinal pigment epithelium (RPE) and photoreceptors. These cells not only have the potential to replace lost cells but also provide trophic support, which may help in repairing damaged retinal structures [27]. Recent clinical studies have begun to assess the safety and efficacy of stem cell-based therapies for retinal degeneration. For instance, iPSC-derived RPE cells have been transplanted in clinical trials, demonstrating initial safety and potential for functional recovery in patients with advanced RP. Additionally, preconditioning strategies for stem cells to mild stressors have been shown to enhance their survival and functional capabilities post-transplantation, potentially improving the effectiveness of these therapies [28]. iPSCs represent a revolutionary approach in regenerative medicine. Derived from somatic cells, iPSCs can be reprogrammed to an embryonic-like state, allowing them to differentiate into any cell type, including those of the retina. One significant advantage of iPSCs is their ability to generate patient-specific cells, significantly reducing the risk of immune rejection [29]. This feature is particularly beneficial for personalized medicine approaches in treating RP. iPSC-derived RPE cells have shown promise in preclinical and early clinical studies, where they have been successfully transplanted into animal models and human subjects. These cells can restore vision by replacing damaged RPE cells and supporting photoreceptor survival [30]. Despite the potential of these therapies, challenges remain in their clinical application. Ensuring the safety of long-term engraftment and preventing tumorigenesis are critical concerns researchers are actively addressing. Ongoing research focuses on optimizing differentiation protocols and improving the integration of transplanted cells into the host retina [26].

Optogenetics and Photopharmacology

Recent advancements in optogenetics and photopharmacology pave the way for innovative therapeutic strategies to restore vision in individuals with retinal degenerative diseases like RP. Optogenetics uses light-sensitive proteins to restore vision by making surviving retinal neurons responsive to light [31]. This technique primarily targets secondary and tertiary neurons in the retina, enabling them to take over the function of degenerated photoreceptors. By utilizing microbial opsins, such as channelrhodopsins, researchers can convert inner retinal neurons into photosensitive cells, allowing these neurons to respond to light and restore some visual function even after the loss of photoreceptors [32]. Recent developments in optogenetics have focused on enhancing the efficacy of these tools. For instance, researchers have developed modified opsins with improved light sensitivity, significantly enhancing visual responses in animal models. These modifications often involve bioinformatics approaches to optimize the opsins' structure for better performance under various light conditions [33]. Clinical applications of optogenetics are also advancing, with ongoing trials evaluating the effectiveness of these therapies in humans. Preliminary results indicate that patients receiving optogenetic treatments can regain some degree of vision, allowing them to perceive light and movement, representing a significant step toward functional vision restoration [34]. In parallel, photopharmacology is emerging as a field that combines pharmacology and photonics to develop light-activated drugs capable of precisely targeting specific tissues or cells. This approach is particularly beneficial in retinal therapies, where precise targeting can minimize side effects and enhance therapeutic efficacy. Photopharmacological agents are designed to be activated by light, allowing for spatial and temporal control over their actions. Recent studies have focused on developing compounds that selectively activate retinal neurons in response to light [35]. These compounds can be used with optogenetic strategies to improve the overall effectiveness of vision restoration therapies. For example, compounds that enhance the activity of remaining retinal cells can be activated by specific wavelengths of light, providing a dual mechanism of action alongside optogenetic treatments [36]. Integrating photopharmacology with optogenetics promises to create more effective therapies for retinal diseases. Future research will likely explore combinations of optogenetic tools and light-activated drugs to enhance visual restoration outcomes. Together, these innovative approaches represent a significant advancement in restoring vision in patients with retinal degenerative diseases, offering hope for improved treatment options and the potential for enhanced visual function in affected individuals [37].

Pharmacological Interventions

Pharmacological interventions for RP are essential for managing the disease and focus on addressing various aspects of retinal degeneration. These interventions primarily aim at neuroprotection, modulation of inflammation, and the development of innovative drug delivery systems [38]. Neuroprotective agents and antioxidants are pivotal in preserving photoreceptor function and slowing degeneration. One of the most extensively studied agents is vitamin A, particularly in the form of vitamin A palmitate. High doses (15,000 IU/day) have modestly slowed RP progression, with a reported reduction of approximately 2% per year. However, long-term safety concerns necessitate regular monitoring of liver enzymes and vitamin A levels [39]. Other promising agents include docosahexaenoic acid (DHA), an omega-3 fatty acid believed to enhance photoreceptor health due to its antioxidant properties. Additionally, carotenoids such as lutein and zeaxanthin are suggested to offer protective effects against retinal degeneration, although more definitive evidence is needed. Calcium channel blockers, like diltiazem, have also shown potential in experimental settings by reducing toxic cyclic GMP levels in the RPE [40]. Anti-inflammatory therapies and immunomodulation are critical in managing the inflammatory processes contributing to retinal degeneration. Corticosteroids, administered through intravitreal injections of agents such as triamcinolone and dexamethasone, have demonstrated effectiveness in treating cystoid macular edema (CME), a common complication in RP patients [41]. Additionally, anti-vascular endothelial growth factor (anti-VEGF) therapies, including bevacizumab, have been used intravitreally to address CME associated with RP, showing positive outcomes in visual function improvement. Carbonic anhydrase inhibitors (CAIs) like acetazolamide have also been explored for their potential benefits in reducing CME, with some reports indicating subjective visual improvements in certain patients [42]. The development of novel drug delivery systems is another promising area of research aimed at enhancing the efficacy of treatments for RP. Viral vectors, particularly adeno-associated viruses (AAVs), are being utilized for gene therapy, allowing for the targeted delivery of therapeutic genes to retinal cells. AAVs are favored due to their low immunogenicity and ability to achieve prolonged target gene expression. Additionally, cell-based therapies involving the transplantation of retinal progenitor cells (RPCs) and mesenchymal stem cells (MSCs) are under investigation [43]. These cells can secrete neuroprotective factors and potentially integrate into the retinal environment to restore function. Another innovative approach is encapsulated cell technology, which involves encapsulating RPE cells to provide the sustained release of neurotrophic factors, thereby protecting photoreceptors and promoting overall retinal health [44].

Technological Innovations

Recent technological advancements in managing RP have centered on retinal prosthetics, artificial intelligence (AI), and wearable technologies. These innovations aim to enhance diagnosis, treatment, and patient experience, providing new hope for those affected by this progressive retinal disorder [45]. Retinal prosthetics, commonly known as bionic eyes, are electronic devices designed to restore partial vision in individuals with severe retinal disease-related vision loss. These devices convert light into electrical signals that stimulate residual retinal cells, enabling patients to perceive visual information. Several retinal prostheses are commercially available, with over 500 patients implanted worldwide. While these devices can improve object localization and environmental navigation, they generally offer limited visual acuity and field of view [46]. Significant challenges include the complexity of the surgical procedure, patient suitability, long-term device durability, and high costs. Ongoing research focuses on enhancing the resolution and functionality of these devices through advancements in materials science and image processing algorithms, including innovations such as smaller electrodes and biomimetic materials to improve integration with retinal tissue [47]. AI is becoming increasingly integral to diagnosing and managing RP, providing several benefits. AI algorithms can analyze imaging data to identify early signs of RP, potentially facilitating earlier intervention. Machine learning models can detect patterns in retinal images indicative of disease progression, enabling timely and targeted treatment strategies [48]. AI also helps develop personalized management plans by analyzing patient data, predicting disease trajectories, optimizing therapeutic approaches, and improving outcomes. Additionally, AI-driven applications enable remote monitoring of patient's visual health, offering real-time data to healthcare providers and enhancing patient engagement with their treatment plans [49]. Wearable technologies and digital therapeutics are emerging as valuable adjuncts in managing RP. Wearable devices, such as smart glasses and other assistive technologies, improve mobility and navigation for individuals with vision loss. These devices often use sensors and AI to provide environmental feedback, helping users avoid obstacles and navigate safely. Digital therapeutics, including mobile applications and software platforms for vision rehabilitation, support patients in adapting to vision loss. These tools typically feature exercises and training programs designed to enhance visual skills and overall quality of life [50].

Challenges and limitations

Developing novel therapeutic strategies for RP is promising, but several challenges and limitations must be addressed to ensure their successful implementation and widespread use. These challenges include safety concerns, accessibility issues, regulatory hurdles, and ethical considerations [51]. Safety is a critical concern when evaluating new therapies for RP. Gene therapies, while targeting specific genetic mutations, may result in unintended effects such as immune responses against the introduced vectors or off-target impacts that could harm healthy cells. Similarly, cell replacement therapies carry risks, including tumorigenesis, immune rejection of transplanted cells, and issues with the integration and functionality of these cells [52]. Additionally, the long-term safety of these treatments remains uncertain, necessitating ongoing patient monitoring to evaluate the durability of treatment effects and identify any late-onset adverse events. Patient variability further complicates safety assessments, as genetic differences among individuals can result in varied responses to therapies [52]. Accessibility and affordability are major barriers to the effective deployment of novel RP therapies. Advanced treatments, particularly gene and cell therapies, are often prohibitively expensive. For instance, Luxturna is priced at over $800,000 per treatment, raising concerns about affordability for healthcare systems and patients [53]. Insurance coverage is another significant issue, as many plans may not cover these costly therapies, leaving patients to shoulder the financial burden. This creates disparities in access, particularly for low-income individuals or those without comprehensive insurance. Additionally, geographic disparities in access to specialized treatment centers can limit availability in rural or underserved areas, exacerbating inequalities in access to cutting-edge therapies [53]. Regulatory hurdles and ethical considerations are also crucial in RP therapies. The regulatory pathways for gene and cell therapies are often lengthy and complex, requiring extensive clinical trials to demonstrate safety and efficacy, which can delay the availability of potentially transformative treatments [54]. Ethical concerns include the manipulation of genetic material, particularly in pediatric populations where informed consent must be navigated carefully by parents. Moreover, there is a risk that research and development may focus on certain genetic mutations or demographics, potentially overlooking rarer forms of RP or underrepresented populations in clinical trials [54].

Future directions and research opportunities

RP management is advancing rapidly and is driven by innovations in precision medicine, ongoing clinical trials, and collaborative research initiatives. One of the most promising developments is the application of precision medicine tailored to individual genetic profiles [8]. This approach leverages comprehensive genomic profiling, facilitated by next-generation sequencing (NGS), to identify specific mutations associated with RP. By understanding a patient’s genetic makeup, clinicians can design personalized treatment strategies that optimize therapeutic outcomes. Additionally, pharmacogenomics, which is the study of how genetic variations influence drug responses, guides adjustments in drug dosages or the selection of alternative therapies, thereby enhancing both efficacy and safety for RP patients [8]. Another critical area of focus is evaluating the long-term outcomes of current clinical trials. The success of these trials will significantly impact RP's future management strategies. Long-term follow-up studies are essential for assessing the durability of treatment effects, safety profiles, and overall patient quality of life [55]. Data from ongoing trials, particularly those investigating gene therapies and retinal implants, will offer invaluable insights into the effectiveness of these interventions over extended periods. Moreover, adopting adaptive trial designs, which allow for modifications based on interim results, can improve the efficiency of clinical research in RP. This flexibility enables researchers to incorporate emerging data, potentially accelerating the development of effective therapies [55]. Collaboration across institutions and countries is crucial for advancing RP research and treatment. Global research networks can pool resources and expertise, enabling large-scale studies that may be impractical for single institutions. Initiatives like the European Union's Horizon 2020 program support collaborative efforts to understand and treat RP internationally [56]. Additionally, establishing biobanks and data-sharing platforms enhances the capacity to conduct multi-center studies, allowing researchers to access diverse patient populations and genetic backgrounds. This collaborative approach can lead to more robust findings and accelerate the translation of research into clinical practice [56].

## Conclusions

RP management has witnessed remarkable advancements in recent years, driven by a deeper understanding of the genetic underpinnings and the development of innovative therapeutic strategies. From groundbreaking genetic therapies and promising cell-based treatments to cutting-edge optogenetics and retinal prosthetics, these novel approaches offer hope for slowing disease progression, restoring visual function, and improving the quality of life for individuals with RP. Despite these exciting developments, challenges remain, including safety issues, accessibility, and the need for further research to validate long-term outcomes. As the field continues to evolve, ongoing collaboration and technological advancements will be crucial in overcoming these hurdles and moving toward effective and personalized treatments. The future of RP management holds great promise, with the potential for transformative therapies that could significantly alter the trajectory of this debilitating condition and bring renewed hope to those affected.
